# What is known about the effects of medical tourism in destination and departure countries? A scoping review

**DOI:** 10.1186/1475-9276-9-24

**Published:** 2010-11-03

**Authors:** Rory Johnston, Valorie A Crooks, Jeremy Snyder, Paul Kingsbury

**Affiliations:** 1 Department of Geography, Simon Fraser University, 8888 University Drive, Burnaby, British Columbia, Canada; 2 Faculty of Health Sciences, Simon Fraser University, 8888 University Drive, Burnaby, British Columbia, Canada

## Abstract

**Background:**

Medical tourism involves patients intentionally leaving their home country to access non-emergency health care services abroad. Growth in the popularity of this practice has resulted in a significant amount of attention being given to it from researchers, policy-makers, and the media. Yet, there has been little effort to systematically synthesize what is known about the effects of this phenomenon. This article presents the findings of a scoping review examining what is known about the effects of medical tourism in destination and departure countries.

**Methods:**

Drawing on academic articles, grey literature, and media sources extracted from18 databases, we follow a widely used scoping review protocol to synthesize what is known about the effects of medical tourism in destination and departure countries. The review design has three main stages: (1) identifying the question and relevant literature; (2) selecting the literature; and (3) charting, collating, and summarizing the data.

**Results:**

The large majority of the 203 sources accepted into the review offer a perspective of medical tourism from the Global North, focusing on the flow of patients from high income nations to lower and middle income countries. This greatly shapes any discussion of the effects of medical tourism on destination and departure countries. Five interrelated themes that characterize existing discussion of the effects of this practice were extracted from the reviewed sources. These themes frame medical tourism as a: (1) user of public resources; (2) solution to health system problems; (3) revenue generating industry; (4) standard of care; and (5) source of inequity. It is observed that what is currently known about the effects of medical tourism is minimal, unreliable, geographically restricted and mostly based on speculation.

**Conclusions:**

Given its positive and negative effects on the health care systems of departure and destination countries, medical tourism is a highly significant and contested phenomenon. This is especially true given its potential to serve as a powerful force for the inequitable delivery of health care services globally. It is recommended that empirical evidence and other data associated with medical tourism be subjected to clear and coherent definitions, including reports focused on the flows of medical tourists and surgery success rates. Additional primary research on the effects of medical tourism is needed if the industry is to develop in a manner that is beneficial to citizens of both departure and destination countries.

## Background

**Figure 1:**
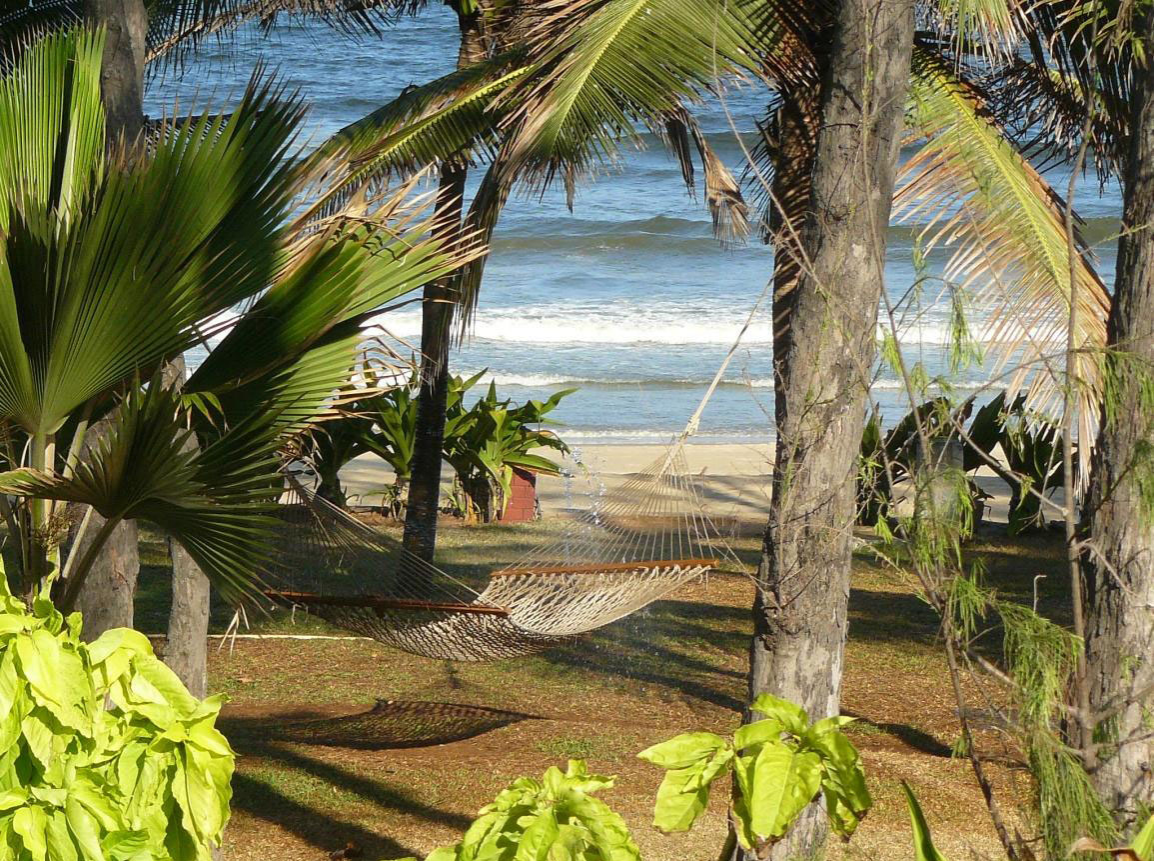
**The tourist element of medical tourism**. Photo credit: authors. This photo shows the beachside view from a high-end resort in Mahabalipuram, India that is affiliated with a hospital treating international patients in a nearby city. Patients can choose to go to this resort during their recovery stay. The photo depicts the popularized image of medical tourism, whereby patients recover in desirable tourist locations after obtaining surgery in LMICs.

Medical tourism involves patients leaving their country of residence outside of established cross-border care arrangements with the intent of accessing medical care, often surgery, abroad [1]. Medical tourists cross national borders to access care, motivated by health service issues such as high costs, lengthy wait times and/or a lack of accessibility in their home systems [2-4]. Popular accounts of medical tourism have emphasized its accessibility and novelty, evoking depictions of recovering from bargain-priced surgeries on beaches [5-7], as shown in Figure 1. For example, a Canadian who had gone to Costa Rica for multiple cosmetic surgeries as a medical tourist said that "It was that best experience that anybody could have because while you are healing, you are in the sunshine...it is absolutely incredible" (n.p.) [8]. A medical tourist from the United Kingdom (UK) who went to South Africa for cosmetic surgery explained that "I went back to the guest house and rested [after surgery]. I didn't have to lift a finger. It was bliss" (p.38) [9]. Accounts such as these contribute to the 'exoticism' of medical tourism, and in many ways shift the focus away from the seriousness of having a surgical intervention while abroad.

Hospitals positioning themselves as leading medical tourism destinations are largely found within lower and middle income countries (LMICs), where favourable exchange rates have given them a competitive advantage in attracting price-conscious international patients [10,11]. This development departs from an earlier pattern of trade in medical services, in which private facilities in high-income countries marketed expensive health services to the global elite [12,13]. Furthermore, trade in medical tourism currently takes place outside of any regulatory framework. This has led some to view it as an expression of profit-driven, commodified private care, and has invited criticisms that it poses as a threat to the equitable delivery of health care worldwide [14-16]. These criticisms are supported by a recent World Health Organization report on health equity that calls for universally available, primary care-oriented health care systems [17]. Specifically, medical tourism requires significant investment in private, for-profit tertiary care infrastructure.

Medical tourism hospitals in LMICs have worked hard to shed the negative images and stereotypes associated with the 'Third World' that are often held by residents of high income countries [18,19]. This includes shifting the perception that goods and services produced in LMICs are of inferior quality [20-23]. The rise of the internet has been integral in this shift, as it has allowed destination hospitals to distribute details about facilities, staff and services to a previously unimaginably wide audience, while maintaining tight control over the presentation of information. For example, websites prominently feature the credentials of physicians, many of whom have trained in prestigious institutions in high income nations [24,25]. Also featured are international safety accreditations that hospitals have secured to further build the confidence of prospective clients [25-27]. Among other things, the promotion of medical tourism via the internet and other mediums has aimed to overcome the reservations international patients might have regarding going abroad for surgery. Meanwhile, numerous risks are inherent in the practice of medical tourism. The possibility of developing embolisms due to flying long periods, especially post-operatively [28], disruptions to patients' continuity of care [29] and the lack of adequate malpractice laws in many destination countries are all risky prospects for medical tourists [30]. These risks have not stopped the steady growth of the industry [31,32].

Coinciding with the growth of the medical tourism industry has been the attention its expansion has received from researchers, policy-makers, and the media alike. Yet, there has been little effort to systematically synthesize what is known about the effects of this practice, even though such a synthesis has the potential to inform research and policy agendas alike. To address this significant knowledge gap, in the remainder of this article we present the findings of a scoping review that sought to answer the question: what is known about the effects of medical tourism in destination and departure countries? Destination countries are conceptualized as those that medical tourists go to for procedures, while departure countries are their home nations. Herein we understand medical tourism to be the intentional pursuit of non-emergency medical treatment outside of a patient's home country that is likely to include a pre or post-operative stay abroad during which some tourist activities may be undertaken. It falls under the larger rubric of 'health tourism' and has a number of distinct streams, demarcated by procedure type. Popular examples are orthopaedic, dental, cardiac, cosmetic, reproductive and transplant tourism [29,33-35]. Formal cross-border care arrangements between countries are excluded from this understanding of medical tourism, as such out-of-country care does not capture the patient-initiated nature of the practice. Also excluded are vacationers seeking emergency care while overseas, ex-patriates receiving treatments in their country of residence and spa vacationers. Distinctions of this nature have been made elsewhere [16].

## Methods

In this article the findings of a scoping review are presented that address the question: what is known about the effects of medical tourism in destination and departure countries? Academic articles, grey literature, and media sources were gathered in a comprehensive fashion in order to answer this question. This is consistent with the scoping review approach, which aims to synthesize what is known about a particular issue across multiple literature types in order to achieve clarity about the state of knowledge and evidence that exists [36]. The synthesis presented here follows the scoping review protocol outlined by Arksey and O'Malley [37]: (1) identifying the question; (2) identifying relevant literature; (3) selecting the literature; (4) charting the data; and (5) collating, summarizing and reporting the results. In the remainder of this section the steps undertaken to complete each stage are outlined.

### Identifying the Question and Relevant Literature

The first step of the scoping review was to hold a team meeting to identify a question that would serve as a useful contribution to scholarship on medical tourism. Following this meeting a search strategy was developed that was tailored to addressing the review question. Team members scanned the published literature on medical tourism to identify keywords relevant to the scoping question. Shown in Table 1, selected keywords probed five categories: (1) focus (e.g., medical tourism); (2) what (e.g., hospital); (3) who (e.g., patient); (4) why (e.g., effects); and (5) where (e.g., destination). Eight types of rationale were identified for the why category (addressing issues such as why the medical tourism industry operates and why patients decide on medical tourism), and keywords were generated for each. Countries known to be departure and destination points for medical tourists at the outset of the review were used to populate the where category.

**Table 1:** Scoping Review Keyword Search Strategy

Focus	What	Who	Why	Where
Medical tourism	Surger*	Patient	Decision making	Destination
Health tourism	Elective surger*	Tourist	Factors	Brazil
	Surgical Procedure*		Decision	India
	Hospital*		Attitudes	Thailand
	Clinic*		Motivation	South Africa
			Destination choice	Indonesia
				
			Tour*	Cuba
			Travel	Mexico
			Vacation*	Philippines
			Adventur*	Singapore
				
			Wait time	United States
			Wait list	Canada
			Queue	
			Speed	
				
			Value*	
			Ethic*	
				
			Privat*	
			Effects	
			Two tier	
				
			Cost savings	
			Affordability	
			Savings	
			Cost	
				
			Motivat*	
			Perspective*	
				
			Distance	
			Quality	

After the keywords were finalized a database search strategy was developed in consultation with a librarian. The strategy was designed to scope the English-language grey, media, and academic literatures in order to find the breadth of sources that would assist with answering the review question. It was decided that combinations of keyword terms would be searched in 18 databases, summarized in Table 2. Academic and media databases required different search strategies. For academic databases, keywords across the five categories were searched using Boolean operators in order to maximize the combinations of terms ultimately scoped (shown as * in Table 1). It was found that some combinations of keywords generated unmanageable results (e.g., generating hundreds of thousands of sources). When such keyword combinations were identified the results were narrowed by the search manager through eliminating the term that had the broadest results. This strategy was found to successfully enhance the relevance of the identified sources.

**Table 2:** Databases Searched for Scoping Review

Database Type	Database	Temporal Period Covered
Academic	Academic Search Premier	1984 - 20/08/2009
	Ageline	1978 - 10/08/2009
	Biomed Central	no recorded start date - 10/08/2009
	Business Source Complete	no recorded start date - 20/08/2009
	Canada Research Index	1982 - 21/07/2009
	CINAHL	1982 - 20/07/2009
	CPI.Q	1988 - 22/10/2009
	EconLit	1969 - 9/08/2009
	Geobase	1980 - 9/08/2009
	Global Health	1973 - 10/08/2009
	Medline	1950 - 9/08/2009
	PAIS International	1972 - 9/08/2009
	PsycINFO	1887 - 10/08/2009
	Sociological Abstracts	1963 - 20/08/2009
	Web of Science	1900 - 10/08/2009

Media	Alternative Press Index	1991 - 20/07/2009
	CBCA Current Events	1982 - 21/07/2009
	Canadian Newstand	1985 - 22/10/2009
	Lexis Nexis	no recorded start date- 22/10/2009

As noted above, the media database search strategy differed from that created for the academic databases. The team first had to identify the geographic scope of the media search. It was agreed that the best strategy would be to search for results pertaining to one country. This strategy was adopted as it was quickly realized that searching all media sources across the globe was not feasible, nor would it be necessary for the purposes of this scoping review. Canada was selected as the country of focus as this is where the research team is based, thus allowing the best access to Canada-focused media databases. Though the media sources included in the review are focused on Canada, they may replicate the types of discussions of medical tourism happening in other known high-income departure nations. 'Health tourism' and 'medical tourism' were the only terms searched in the media databases listed in Table 2. In addition, specific North American sources known to cover Canadian health services stories were searched within the Lexis Nexis database (namely the Globe and Mail, New York Times, Associated Press, Time inc., Washington Post, magazines, Toronto Sun, Toronto Star, CBC News). Across the searched academic and media databases, sources of all types (e.g., business briefs, research articles, newspaper editorials, industry reports) deemed relevant were retrieved. Retrieved sources were stored using the Refworks bibliographic management program.

### Selecting the Literature

Titles and abstracts of sources identified by the search strategy were independently reviewed by team members in batches in order to select literature for inclusion in the scoping review. After completion of the independent review, team members met and sought consensus regarding whether a particular source was to be read in full. Consistent with the scoping review process, *post hoc *inclusion criteria were developed at this point [37]. Three bases for exclusion were identified, namely that there was either: (1) no focus on medical intervention, such as sources that dealt with health tourism more broadly (e.g., international travel to healing spas); (2) an exclusive focus on 'reproductive tourism' or 'transplant tourism', as the medical intervention (if any) in such cases is not restricted to the international patient and thus raises separate considerations; and/or (3) an overly general focus on international trade in health services or cross-border care, where there was no explicit reference to medical tourism. Any sources not in English were also excluded at this point. In instances where there was disagreement between team members as to whether or not a source should be reviewed in full, the source was discussed until consensus was reached. Such disagreement was minimal as the level of agreement among team members was high from the outset.

Once the title and abstract review was complete, included sources were reviewed in full. It should be noted that abstracts for media sources were not available and so all were reviewed in full. The reference lists of sources accepted for full review were hand searched and relevant sources not identified by the initial strategy that were deemed relevant to the review question were also reviewed in full. The exclusion criteria developed at the title and abstract review stage were again applied during full review. One additional criterion was added: if no 'data' (i.e., information points that contributed to answering the scoping question) were extracted from a source it was excluded. Every source identified for full review was read by two team members who were assigned by the search manager. Sources were reviewed in batches. As each batch was completed, the team met to make decisions regarding the inclusion or exclusion of sources. As with the title and abstract review stage, the level of agreement was high and any disagreement was resolved through seeking consensus among the entire team after discussion.

### Charting, Collating, and Summarizing the Data

A spreadsheet securely hosted online and used by all team members was created to chart the data extracted from sources reviewed in full. Details regarding study design and sample (if relevant), publication information, and data pertinent to the scoping question were recorded in the spreadsheet. A series of meetings were held in order to discuss the extracted data and gain an overall perspective on the issues emerging from the literature. After this the lead author reviewed the extracted data and determined the overall themes that best characterized what had been gleaned from the sources reviewed. The identification of themes in the reviewed literature deemed relevant to the scoping question is an essential part of the charting process [37]. Team members reviewed the themes to confirm the interpretation that had been generated. The extracted data in the spreadsheet was next colour coded according to theme. This was done to assist with organizing how the findings of the scoping review would be reported. The final step was for team members to work together in order to identify key knowledge gaps emerging from the reviewed sources that have direct relevance to the scoping review question.

## Results

**Figure 2:**
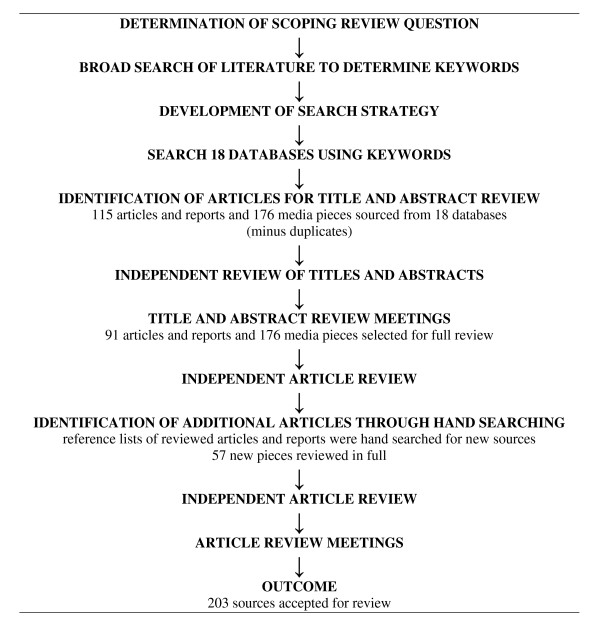
**Scoping Review Search Strategy and Results**.

As shown in Figure 2, 291 sources were extracted for review from the 18 databases searched. A further 57 were found from hand-searching reference lists. Following the partial (title and abstract) and full reviews of these 348 sources, 203 were ultimately accepted for inclusion in this scoping review (a complete list of the sources included can be obtained by contacting the lead author). When compared with other methods of knowledge synthesis, such as systematic reviews, this is a very large sample with which to work. However, given that the goal of a scoping review is to characterize the breadth of knowledge surrounding a fairly open question [37], the size of the sample offers confidence that the review comprehensively and exhaustively captures what is known about the effects of medical tourism in destination and departure countries.

Of the 203 sources included in the review, almost all were speculative in nature. A very small number (*n *= 6) were empirical studies reporting on primary data, while the majority (*n *= 107) of those included were from popular media sources. The remaining 91 are a mixture of reports, legal reviews, commentaries, editorials and business briefs. Most sources offered a perspective on medical tourism from the Global North, especially from the United States, focusing specifically on the flow of patients from developed nations to LMICs in the Global South. This significantly shaped discussion of the effects of medical tourism on destination and departure countries. Details of these effects, as gleaned from the reviewed sources, are broadly characterized by five crosscutting themes. These themes frame medical tourism as a: (1) user of public resources; (2) solution to problems; (3) revenue generating industry; (4) standard of care; and (5) source of inequity. As can be seen from these themes, the focus of discussion is on system-based effects rather than individual-level ones. In the remainder of this section we elaborate on these themes, exploring their positive and negative effects in both destination and departure countries.

### Medical Tourism as a User of Public Resources

In discussions of the relationship between medical tourism and its influence on the allocation of public resources in destination countries, the practice has regularly been treated as a threat to the equitable use of public funds. Most commonly discussed has been India's rapidly growing private medical tourism industry, which has been aggressively cultivated with the aid of government funds [27,38-42]. Government subsidies have been provided in the form of the provision of public land, corporate tax breaks and reduced tariffs on imported medical equipment for use in private hospitals serving medical tourists [38,39,42]. Though these private hospitals are required to provide some health services for impoverished domestic patients, there have been reports that these regulations are token requirements that regularly go unmet [43]. The use of public resources for medical tourism also extends to nations' publicly-financed health care services, with countries such as Singapore and Cuba opening public hospitals to privately-financed foreign patients [21,44-47]. A less direct form of public subsidization, the hiring of physicians trained in public education systems by private medical tourism facilities is another example of a potentially inequitable use of public resources [38,48]. Furthermore, physicians in LMICs who might normally practice in resource-poor environments can instead treat high-paying international patients, thereby gaining access to advanced technologies and superior facilities while receiving a higher wage [38,49]. There has been some direct rebuttal of this concern by those who note that many doctors working at medical tourism facilities in LMICs were educated in high-income countries and are, in fact, returning to practice in systems they had previously left behind [11,50].

Medical tourists may face major medical complications upon return to their home countries or require some form of post-operative care. A number of sources reviewed raised the issue of this care being a diversion of public resources, particularly when a medical tourist's domestic system is partially or fully funded through public financing [1,51]. For example, an Australian study demonstrated that treating an acutely infected total joint arthroplasty that had been performed abroad resulted in costs that were nine times what would have been spent had the patient received the procedure in a domestic hospital [52]. While this case vividly depicts the possible public costs of post-operative care stemming from medical tourism, the authors note that even with a 5% failure rate, the cost savings potential offered by medical tourism are so substantial that the Australian health care system would save public money by allowing patients to pursue the operation overseas and receive reimbursement [52]. Patients in other countries with publicly-funded health care systems may also be reimbursed by their governments for pursuing some elective surgeries abroad, likely lowering cost barriers to engaging in medical tourism. For example, a very small number of Canadian medical tourists have been reimbursed by their provincial public Medicare systems upon returning home if, prior to departure, they were able to demonstrate that the care being sought was medically necessary and unable to be provided in a timely manner in Canada [31,53,54]. However, in such cases medical tourism becomes a viable option only for those who can afford to first pay out-of-pocket.

### Medical Tourism as a Solution to Health System Problems

Development of the medical tourism industry in LMICs carries with it a number of potential benefits that work to address some existing health system problems related to infrastructure development and retention of health human resources. Foremost amongst these is its purported capacity to spur both local and foreign investment into health care infrastructure [21,41,42,55-57]. Use of such infrastructure may not be limited to medical tourists, thus benefiting local patients [19,48,58,59]. In fact, most hospitals providing services to international patients are primarily dependent on locals for the bulk of their business [10,11]. Investment into more advanced medical services in LMICs can also encourage patients who would otherwise travel abroad for care to get care at home, thus keeping capital within the country [58]. Another direct benefit resulting from the new infrastructure necessary for attracting medical tourists is the creation of long-term, high skilled jobs necessary for a strong tertiary health care system [1,60]. It has also been suggested that the financial (e.g., high salaries) and technical (e.g., high technology work environments) incentives for doctors practicing in medical tourism can slow, or even reverse, the migration of locally trained health human resources abroad [1,11,19,61]. Another often cited point is that medical tourism draws hard currency into LMICs, particularly from higher income countries [1,5,10,14,62].

Medical tourism is depicted as a potential solution to an array of problems facing health care systems in patients' home countries, which can be most briefly summarized as those of the cost and affordability of care, wait times and access to medical facilities and treatments. The most commonly discussed among these is the issue of unaffordable care. Medical tourism is often presented as providing an option to patients in countries where medical care is prohibitively expensive [63,64]. An extension of this view is the potential for a vigorous medical tourism industry to drive down domestic costs and encourage price transparency by introducing competition into countries with captive private markets, such as the United States [63,65]. In countries with publicly financed health care systems, most notably the UK and Canada, medical tourism has been discussed as a solution for reducing long surgical wait lists [3,31,55,59,66]. For citizens of LMICs, it can offer access to needed care abroad that is simply unavailable at home due to inadequate medical infrastructure [10,67]. For these patients, receiving care in another country may be their only option, and contrary to the levity of the label 'medical *tourism*', it may prove to be an impoverishing experience [68,69].

### Medical Tourism as a Revenue Generating Industry

The medical tourism industry is a lucrative source of hard currency for destination countries. Its annual growth potential has regularly been estimated above 25% for LMICs such as India, Malaysia and Thailand [2,18,70]. However, estimates of patient and currency inflows and their potential for growth are wildly varied, as definitional issues confound the already difficult attempt to determine the scale of the industry [2,3,71,72]. Examples of this include: estimates for the entire global medical tourism industry have been as low as 60,000 patients annually [2], while estimates of the annual American outflow alone have been as varied as 50,000 to 500,000 [63]. Perhaps the best example of speculative reporting has been the widely cited Deloitte report, which estimated an American patient outflow in 2007 at 750,000 and projected its increase to rise to 10,000,000 by 2012 [19]. Regardless of the actual numbers, it is generally agreed that the industry is growing rapidly in many Asian and South American nations, and is poised for significant growth in countries with the necessary human and technical resources [1,10,58,73,74]. India and Thailand's introduction of expedited medical visas is a sign of deep commitment to facilitating trade in medical tourism [40,48,70]. Thailand has gone so far as to try and negotiate the portability of public insurance between countries in the region to enhance the flow of inbound international patients [75]. Medical tourists are particularly valuable because of the money they invest in the local economy when compared with traditional tourists. For example, it has been estimated that medical tourists visiting Asia spend over twice as much as traditional tourists [76]. Cuba is an example of a country that established itself as a destination early on in the development of medical tourism, and it has built its industry into a revenue generator that has been used to fund its public health care system [45]. There has been a similar instance of providing care at a premium for foreign patients and using the difference to subsidize care for locals at a non-profit hospital in India [77].

Accompanying revenue gains in destination countries have been associated revenue losses in medical tourists' home countries. While the scattered estimates of international patient flows calls into question the reliability of estimates that find the United States currently losing billions of dollars annually to medical tourism [78,79], it is only logical to assume that every patient leaving one health care system for another is taking their capital elsewhere. In response to the travel and care coordination needs of medical tourists, a cottage industry of brokerages has sprung up within patients' home countries. These brokerages coordinate the necessary travel, medical, accommodation and holiday arrangements for medical tourists, in exchange for fees from patients or a commission from the hospitals on a per-referral basis [3]. These brokerages have rapidly expanded to fill a niche and are often based in patients' home countries, thus further spreading the global reach of industry profits. This expansion has been facilitated by the spread of the internet, which has made medical tourism an increasingly accessible option and has helped Canada alone support more than 20 different brokerages [14].

### Medical Tourism as a Standard of Care

A number of changes to standards of care have accompanied the development of medical tourism in destination countries. Perhaps the most noticeable has been the adoption of international accreditations that are modeled on Western standards [26,56,80]. Foremost amongst these is hospital accreditation by the Joint Commission International (JCI), an offshoot of the primary body responsible for accrediting hospitals in the United States. The development and implementation of JCI has been credited with helping medical tourism to flourish by guaranteeing a standard of care comparable to that found in American hospitals in accredited hospitals [65]. This means of securing the trust of Western patients carries with it the potential for American standards to override local values and approaches to providing health care [15,80]. However, the accreditation of an American-style quality of care does not extend to assurances of American-style liability. Most LMIC destination countries have limited malpractice laws and insurance requirements, leaving medical tourists with few opportunities for recourse should procedures go awry [1,19,27]. Another concern is the use of Western place aesthetics by medical tourism facilities, a phenomenon that has led to the displacement and homogenization of previously non-Western care spaces [15,21]. This displacement includes designing hospital lobbies to look like shopping malls and patients' rooms to look like upscale hotels. Medical tourism hospitals are also able to deliver very high nurse-to-patient ratios, higher than those found in wards designated for local patients, due to the low costs of labour in LMICs [7,25,35]. Medical tourists are also typically given the option of recovering at nearby resorts, offering amenities not replicated in the hospital environments that non-international patients may be required to stay in during recovery [1,55,81,82].

Medical tourists returning to their home countries may bring with them altered conceptions of appropriate standards of care. Exposure to nurse-to-patient ratios that far exceed norms in most systems, hotel-like care spaces and the ability to choose one's physician from a website may result in patients demanding similar 'consumer as king' treatment at home [1,83]. Expectations such as these are tied to the treatment of health care as a commodity, and may be especially deleterious for overburdened publicly-funded health care systems. More specifically, patients returning from abroad may wish to see elements of their privately-funded care instituted within their home systems [84,85]. Medical tourism can also create a sense of limitless options for potential patients. If they find themselves unhappy with local options, the seemingly boundless variety of willing facilities and professionals available internationally holds with it the promise for effective treatment somewhere. This can encourage patients to treat medical tourism as a viable solution to medical problems, even if their prognosis is beyond treatment [68].

### Medical Tourism as a Source of Inequity

Medical tourism has regularly been accused of exacerbating health care - and ultimately health - inequities in destination countries. One of these charges is that it exacerbates 'brain drain' within destination countries. The higher wages and advanced technolologies available at facilities offering medical tourism act as a lure for health care providers working in more modest facilities [40,41,48]. Because medical tourism facilities are primarily urban, this process also hastens the internal migration of health care providers from rural areas into cities, thereby enhancing rural deprivation [56,75,86]. If the medical tourism industry achieves even a fraction of the flows of patients envisioned by early commentators, this could ultimately lead to locals being priced out of their own health care system, as demand from foreign patients can drive up the costs of providing care for everyone [14,61,66,87]. The financial gains medical tourism offers LMICs has worked to shift health care investments into expensive, high-technology tertiary care that benefits a limited number of patients for the high cost outlay [39,88]. This is especially inequitable given the large proportion of the population across LMIC destination countries for whom basic medical services are prohibitively expensive or simply unavailable [3,40].

Medical tourists traveling from LMICs may end up exhausting their finances to access care, impoverishing themselves and their families, and thereby creating or exacerbating inequities [68,69]. As long as the option for treatment exists elsewhere, departure countries lacking medical services may feel little pressure to address shortages, pushing citizens to risk material wellbeing in the pursuit of needed care [68,69]. When patients from countries with strong publicly-funded health care systems engage in medical tourism they undermine the ethos that has allowed these very systems to develop and survive [89]. They do this by effectively introducing a second tier of care available only to those with the ability to pay, an option that runs contrary to the provision of exclusively publicly-funded health care. Their actions extend further than the appearance of inequity, as each medical tourist puts his/her support behind the private provision of health care and a conception of health care as a commodity [1,39,44]. This also applies to countries lacking in public health care, as the affordability of medical tourism works to neutralize pressure for reform and the equitable distribution of resources [90].

## Discussion

The results of this scoping review make it clear that what is currently known about the effects of medical tourism is minimal and that speculation abounds. Medical tourism is a phenomenon born out of a steadily globalizing economy, and is an expression of private, for-profit health care. The potential inequities this model of care can cultivate in both departure and destination countries can work to damage the provision of publicly-funded care where it is established and inhibit its development where it is not [14,16,91,92]. Conversely, the potential for medical tourism to operate in a mutually beneficial and equitable manner exists. This, however, requires oversight and regulation that guarantees meaningful compensation to the citizens of countries that offer their medical services to international patients [48,59,60].

What is known of medical tourism's effects is most effectively summarized by the five themes identified and reviewed above. These themes have particular implications for three global spheres of activity common to both departure and destination countries, namely those of their: health care systems, health and social policies and involvement in the medical tourism industry itself. These implications are expanded upon in this section, with consideration also being given to knowledge gaps identified by, and the limitations of, this scoping review.

### Implications of the Effects of Medical Tourism

The sources reviewed indicate that medical tourism has, and will continue to have, significant effects on the health care systems of both departure and destination countries. For health administrators in patients' home countries, the lack of surveillance and monitoring of the practice of medical tourism can result in unaccounted for 'leakage' of patients outside of their jurisdictions [27,32]. In systems that ration care, this negatively impacts the ability to accurately anticipate and distribute health care resources effectively. This could result in a situation where a health care system is perceived to be unresponsive to the needs of returning medical tourists due to inadequate resources being allotted for follow-up care. Medical tourism also works to introduce and normalize profit motives within the 'cultures' of the health care systems it engages with. For example, there are accounts of this approach to care resulting in the substitution of clinical factors with financial ones in treatment decision-making amongst medical tourists [52,75]. This approach to decision-making may also negatively impact the local population in destination countries should a culture of compassion and necessity amongst health care professionals become supplanted by one of financial opportunism and pragmatism.

Countries hoping to capitalize on the ability of medical tourism to spur investment into their domestic health care infrastructures in the short term may find themselves becoming structurally dependent on foreign sources of investment and income well into the future. This dependency can lead to the entrenchment of inequitable modes of health care delivery [30,93]. In countries that have signed over access to health care services under the international General Agreement in Trade in Services, this could bring about the permanent involvement of foreign parties with no vested interest in the well-being of local populations into their health care landscapes [14,75]. Finally, the argument that destination countries should view investment into technology-intensive infrastructure for medical tourists as beneficial is undermined when the types of services being offered by hospitals are contrasted with the pressing health care needs of the local population. This contrast is all the more troubling when public money that could be used for wide-reaching, inexpensive primary health care initiatives is used to incentivize private investment into expensive tertiary care with a more limited impact.

Medical tourism has been likened to the 'wild west' with regard to the regulatory environment it occupies [12,27]. This has resulted in what appears to be a lack of any discernable momentum to develop policy responses that can help to improve patient safety measures or guide the development of the industry in an equitable and ethical manner. As it stands, medical tourism is a largely characterized as an inequitable trade practice. LMIC destination countries offer essential services and skills that are needed for their own populations to foreigners with no vested interest in the success, failure or fairness of the systems they are using [39,46]. There is, however, much room for improvement in such a young and unregulated industry. As an example, equitable buying guidelines could be established to assist patients and providers with better assessing the potential impacts of the medical tourism industry. Equitable buying guidelines have been developed and implemented in other global industries that have been implicated in inequitable business practices, and could be mirrored in the medical tourism industry [94,95]. Likewise, patient safety could be greatly improved by developing stronger malpractice laws in destination countries and better informational and care coordination tools amongst departure countries. Implementing international standards for surveillance and monitoring of medical tourism would also allow for better care and planning by governments of both departure and destination nations.

Important questions are raised by this scoping review about the current organization of the medical tourism industry. The lack of regulation and standards amongst medical tourism brokerages have allowed for anyone to establish a brokerage [96-98]. For medical tourists who lack the confidence, knowledge or skills to arrange a medical tour on their own, private business owners who lack technical medical knowledge while having a financial stake in securing patients are, at the same time, their gatekeepers to care [3,11,14,46]. While there is no evidence in the literature reviewed suggesting that there are problems with predatory or irresponsible business practices amongst medical tourism brokerages, regulatory voids around essential services set the stage for dangerous business practices that would be better pre-emptively managed.

Some medical tourism hospitals have ardently sought accreditation by trusted third party assessors such as JCI. This process is an indication that there is a willingness to self-regulate within the industry, and it raises the question of whether or not existing accreditation schemes should be altered or new schemes should be created that can assure consumers of equitable trade practices. Examples of these assurances could include meaningful levels of pro bono service for locals by medical tourism facilities, the creation and maintenance of primary health care clinics in under-served regions and other such measures to encourage health equity in destination countries.

### Knowledge Gaps

The scoping review process demonstrated that there are major gaps in what is known about the effects of medical tourism. Most broadly, there is currently a very limited body of empirical research on the topic. In general, the reviewed sources revealed that *initial *estimates and ideas about medical tourism were heavily cited and recycled until they became treated as facts, both within and outside of scholarly publications. This suggests that existing sources must be approached carefully, lest misrepresentation be perpetuated further. The dearth of research-based knowledge about medical tourism does, however, provide a 'green light' for the development of new research into the phenomenon. The need to develop research is pressing given the number of potential impacts medical tourism poses for the equitable delivery of health care in departure and destination countries alike, along with the other effects this practice holds. In effect, research from across the social and health sciences is necessary to begin to properly document and meaningfully explore this trade practice. In the remainder of this sub-section a number of specific knowledge gaps are expanded on so as to identify possible 'ways ahead' for researchers interested in exploring medical tourism.

First and foremost, there is no reliable hard data on patient numbers, flows, treatment types and success rates. As it stands, there are conflicting estimates of patient numbers, with magnitudes of difference between them that likely stem from definitional issues of what comprises medical tourism. Success rates are reported by some facilities, but without an independent auditor or the inclusion of long-term success rates after medical tourists return to their home countries, these are of little value. This contrasts sharply with the glowing reports of treatment success rates in some business briefs, reports and popular news media that have failed to acknowledge the purely speculative and self-reported nature of the numbers at this point. Research is needed that can produce reliable understandings of the scope and volume of the practice of medical tourism in a way that is comparable across facilities and countries. Such research could also offer some of the first insights into important differences between medical tourists based on factors such as age, gender, citizenship, and health status, where such differentiation was simply not considered by the sources included in the scoping review. Destination countries that have introduced medical visas serve as a logical place to begin collecting this valuable data. The continuing failure of departure countries to introduce monitoring practices, especially using standardized units, makes it difficult to collect corresponding data at the other end of the medical tourism trail.

Legal and regulatory issues related to medical tourism must be expanded upon by researchers. As it stands, reviewed sources only managed to capture legal perspectives from the United States. While these sources likely offer a useful template for legal issues surrounding international trade between high income nations and LMICs, legal perspectives from other nations, especially those of destination countries, are needed to help to further clarify issues of malpractice and other legal dimensions of the industry. Regulatory frameworks are often cited as a necessity to ensure the safety of medical tourists, but sources that seek to realistically outline what these regulations would look like and how they would operate are absent. Meanwhile, these frameworks are necessary if the continuing expansion of the industry is to adopt safe and equitable approaches to organizing and delivering care.

Finally, issues of equity have largely been ignored in the popular, legal and business literature surrounding medical tourism, with reports instead focusing on the novelty of the industry or low prices available to potential patients. Within the academic literature, health equity has been more of a 'hit or miss' issue. For example, medical tourism has been consistently cited as a solution to both the problems of wait times and cost in departure countries by enhancing access to needed care, despite there being no evidence of it impacting either of these factors at the system level. In relation to this same point, much less attention has been given to understanding how such an approach to addressing system problems exacerbates inequities among residents of departure countries through enabling access to care abroad only for those who can afford it. Future research must make an effort to clarify what impacts medical tourism trade practices and financing arrangements are having on the delivery of health care within departure and destination countries, and at the very least provide the basic context for understanding how health care is currently arranged within them.

### Limitations

Despite rigorous measures such as using multiple reviewers on all sources and seeking the guidance of a librarian in designing the search strategy, two major limitations are evident in the scoping review process employed. First, only English-language sources were considered for inclusion. Given the global nature of medical tourism, it is unlikely that all relevant literature is available in English. Second, media sources reviewed were restricted to those from Canada and a small number of major North American magazines and newspapers with relatively large Canadian audiences. This restriction was a necessary one, as the sheer volume of irrelevant popular literature to initially sort through in a non-restricted search would have rendered the scoping review unmanageable. Though focused on Canada, the media sources that were ultimately included provide a thorough illustration of the kinds of discussions about medical tourism being held locally, regionally and nationally in other high-income departure countries such as the United States and the UK.

## Conclusions

This scoping review has identified five themes that effectively synthesize what is known about the effects of medical tourism in destination and departure countries. First, medical tourism can be understood as a user of public resources. This includes consuming public health care resources in destination countries through redirecting them to the private sector, and in departure countries through the provision of follow-up care for medical tourists. Second, the practice of medical tourism can be seen as offering solutions to problems. It has the potential to aid in the development of health care infrastructure in destination countries while reducing wait times and costs for care for residents of departure countries. Third, medical tourism is also a revenue generating industry. This industry can generate revenues for destination countries as a form of health services trade. Conversely, it can result in a net loss of capital to departure countries. Fourth, medical tourism can be seen as setting a standard of care. By seeking accreditation, destination countries may develop a Western-oriented standard of care, including in facility aesthetics. Due to low labour costs in destination countries, medical tourists may develop expectations of standards of health human resource provision that are unaffordable, and therefore unattainable, in high-income departure countries. The process of medical tourism can also contribute to the commodification of health care and a perception of the patient as consumer. Finally, the cumulative effects of medical tourism position it as a source of inequity. Within destination countries, it can contribute to an internal brain drain of trained medical workers from rural to urban areas and from the public to the private sector. Medical tourists can face a significant drain on their own financial resources and, by engaging in travel abroad for medical services, they may contribute to a loss of impetus for reform of their home health care systems.

The findings of this scoping review indicate that medical tourism is a very important phenomenon given its effects on the health care systems of departure and destination countries alike. Various commentators point to both the potential for negative and positive effects of medical tourism, marking it as a contested phenomenon. Without regulation, the potential positive effects may not be realized and medical tourism may contribute to a harmful global commodification of health care. These regulations must be developed, but the ability to do this is hampered by significant gaps in our knowledge of the effects of medical tourism, as identified by this review. To address these gaps, data regarding medical tourism must be subjected to clear and unified definitions of the phenomenon, flows of medical tourists and success rates of their surgeries must be clearly documented, and additional primary research into the impacts of medical tourism must be mounted. Until these research gaps are filled, medical tourism is likely to remain an increasingly visible and practiced global phenomenon, subject to much conjecture but little regulation or understanding.

## Abbreviations

JCI: Joint Commission International; LMIC: Lower and middle income countries; UK: United Kingdom

## Competing interests

The authors declare that they have no competing interests.

## Authors' contributions

RJ led the identification of themes and writing of this manuscript. All authors contributed to the design of the scoping review, reviewing sources, and assisting with data management and interpretation. All authors provided feedback throughout the drafting of this article and have read and approved the final manuscript.
